# The Present Status of Electricity in Medicine

**Published:** 1900-10

**Authors:** William Benham Snow

**Affiliations:** Atlanta, Ga., Late Instructor in Electro-Therapeutics and Nervous Diseases in the New York Post-graduate Medical School and Hospital


### THE PRESENT STATUS OF ELECTRICITY IN MEDICINE.

BY WILLIAM BENHAM SNOW, M.D., ATLANTA, GA.,

Late Instructor in Electro-Therapeutics and Nervous Diseases in the New York Post-graduate Medical School and Hospital.

Since the first accidental discovery by Galvani, or Galvani's wife, that something caused contraction of a dead frog's legs, and that something was called electricity, the medical profession has hopefully made use of it to effect some therapeutic result.

Various views have existed, such as "electricity is life," or an essential element of life. Not, as is more probable, a force in nature, variously affecting or quickening life.

To it have been relegated all manner of incurable and obscure diseases after every other remedy has failed. The unknown, to affect an unknown diseased condition, in an unknown manner, has been used to obtain a happy result, and so it is to this day. It is the *dernier ressort* of all incurables, and to be sure no favorable result is expected, but something must be done, and the word is given, "try electricity." How many to-day know how to "try electricity?" Ninety-nine of every one hundred *candid* physicians admit that they know very little, if anything, about it. This is not strange, when we take note that the subject is treated in the most indifferent manner in the curriculum of all but few of the medical colleges of the country.

Under these conditions it is not surprising that so many neurologists refer to it under the head of "suggestive therapeutics," for without scientific application, employing crude and ineffective apparatus, it does become suggestive. What has contributed most to this deplorable state of knowledge of electro-therapeutics is the fact that the masters of the art have relied upon two forms of current, one producing marked and valuable local effects but otherwise of doubtful efficacy, and the other not possessed of energy sufficient to produce other than muscular contraction, moderate local sedation and reflex action induced by peripheral stimulation. These effects have not produced satisfactory results in the hands of the mass of physicians because they were unable to use them intelligently, while experts have found them disappointing.

There are two qualities of every electric current upon which the character of the action depends—the amperage, or quantity, and the voltage, or pressure. The amperage of a battery of galvanic cells will be relatively large and the voltage insignificant, while the interrupted (Faradic) current will have a very small amperage and much higher voltage. The currents of the static machines are currents of infinitesimal quantity and extremely high voltage.

The distinctive actions of the various currents always depend upon varying proportions of quantity and voltage, and the quality of current will depend upon the arrangement and construction of the cells generating the current, the length and size of the wire in the battery, of the interrupted (Faradic), and the number and size of the revolving plates of the static machines, together with other manipulations of the parts of the battery or machine.

The *constant current* (better known as the galvanic, but a term already in disfavor), from its nature possesses polar qualities which make it useful in many operations where the destruction of tissue, or other local alterative effects are indicated, such as the removal of angioma and other facial blemishes, operations upon hemorrhoids, urethritis, carcinoma, lupus, tubercular glands, uterine fibroids, strictures of the urethra, etc. There are many who question the wisdom of these procedures, and, on the other hand, there are others who, understanding the technique, are very successful in the employment of them. That there is a large field for the employment of this current in the procedures of electrolysis, metallic electrolysis, cataphoresis, the action of the negative pole upon scar tissue, and its effects in inducing peristaltic action in acute or chronic obstructions of the lower bowels, cannot be denied. It is questionable, from its peculiar polar effects, if it is not objectionable where other than destructive or alterative local effects are sought, and it is certain that the other currents are preferable for all administrations where tonic, nutritional, sedative and derivative effects are desired. The interrupted current of the Faradic battery acts as a local stimulant to muscular contraction, and is mildly stimulating or sedative. It may also in a feeble way promote local nutrition. These effects, except painful muscular contractions, can only be obtained from a modern battery having thousands of feet of fine wire in the secondary over a primary coil of very coarse wire, and a device providing for very rapid interruptions. The batteries selling at from ten to fifteen dollars are of very little therapeutic value, and no Faradic battery should be considered valuable except at the bedside when electro-static currents cannot be employed.

The sinusoidal current is a very agreeable current, but in this respect not so pleasing as the static wave current, while in potency it bears no comparison. The great frequency, high potential currents, generated by the machines of D'Arsonval, which stand so high in favor with the profession abroad, are currents of so large amperage that 1,500 volts is the maximum safe for administration, while 1,000,000 volts, about the maximum capacity of a powerful static machine, will not endanger the life of a child. To this it may be added that there are few reasons why the former may be more efficacious, and many are the advantages of the latter. We therefore prefer and advocate the American electro-therapeutics of the modern static machines made in this country, of which no equal is produced abroad.

Static administrations include several forms of application—static electrization, breeze, sparks, and spark-gap currents of Morton—each of special therapeutic value, but generally considered under the terms, Franklinization or electro-static treatment. Considered as a whole the *physiological actions* of electro-static administration are as follows:

I. THE CONSTITUTIONAL EFFECTS.

1. Marked lowering of arterial tension.

2. Lessened frequency of heart's action, with increase of its force and the volume of the pulse.

3. Increased oxidation and metabolic activity, marked by body warmth, deepened inspiration, increased production of $CO_2$, increased elimination of solids with the urine, and marked functional activity of the organs of secretion and excretion.

4. Diminution of nervous irritability and marked sense of drowsiness and fatigue if treatment is prolonged. Administrations of powerful currents are well borne for one-half hour, by well-developed patients, but a milder current (measured by one-inch spark-gap with wave current), administered for fifteen minutes, will be sufficient for others.

5. It has been demonstrated that patients receiving treatment for from one to two hours daily (two administrations) for periods of months gain body weight, become less anemic, bowels continue regular, digestion good, and muscular tone is preserved without recourse to drug administration and while taking no bodily exercise. We may then truthfully add that electro-static administrations are absolutely harmless and a most active tonic, promoting nutrition and inducing general functional activity.

II. THE LOCAL EFFECTS are many, and depend upon the technique and form of administration for results.

1. It *diminishes* local *hyperemia* or *congestion*, when not due to a specific poison or necrosis. (It is not demonstrated that static applications affect germ life except the ozonizing effect of the breeze or the brush discharge from the wood electrode when locally applied.)

2. It sedates pain and nervous irritability.

3. It overcomes muscular spasm and contraction when properly administered. (Sparks or wave current.)

4. It excites muscular contraction, induces physiological tetanus, and when prolonged produces fatigue and relaxation by exhaustion. (The wave current.)

5. *Glands* and *end organs* of parts beneath electrode, or to which sparks, breeze, or brush discharge are applied, are stimulated to greater activity, marked by profuse sweating, and often by diminution of swelling during the administration.

6. The *brush discharge* from the wood electrode is a *rubefacient*, the counterirritant effect surpassing a sinapism.

7. The *brush discharge* is undoubtedly a mild *antiseptic*, due to ozone generated at the points of contact.

*The therapeutics* of electro-static administration verify in a most forcible manner all that the physiological actions would imply; but to succeed requires painstaking care in the treatment of each case, time enough to attain the desired result, frequency of application, which will bridge over or maintain a degree of favorable change or advantage between sittings, and, what is of still greater importance, the technique applicable to each case.

The constitutional effects have been demonstrated in very many cases, when patients have been under treatment for affections of a local character, as well as in those suffering from nervous disorders, such as neurasthenia, various forms of paralysis, locomotor ataxia or rheumatoid arthritis. Neurasthenia represents such a variety of functional disorders that an agency which succeeds in relieving all of the symptoms will deserve a high rank as a restorative. No agent after removal of the cause will contribute so much to relieve neurasthenia as static electricity. By restoring a general functional activity, all emunctories are opened, secretion and excretion restored, the bowels become regular, digestion and appetite improve, nervous irritability and painful conditions are relieved, normal sleep is restored, and the patient recovers. The writer has seen this train of sequence so often repeated that he speaks with confidence.

In locomotor ataxia, as the pains and nervous irritability are gradually relieved, the patients gain body weight, anemia becomes less pronounced, nervous resistance against the progress of the disease increases, there is muscular development with gaining strength and consequent improvement in coordination. There will be some diminution of anesthesia, more or less restoration of impaired functions, and in very many cases positive arrest of the disease. There are already enough such cases on record to substantiate this statement, despite contradiction. *Rheumatoid Arthritis*, another, until recently, considered incurable disease generally incapable of arrest, probably of nervous origin, has now yielded in many cases to static treatment. In cases of not more than two years' standing, where the joint structures have not suffered change, the prognosis is good; in others it may be arrested and the suffering much relieved. The writer presented several cases before the Clinical Society of the New York Post Graduate in October, 1899, published in the *Post Graduate* of November of that year. In all functional diseases due to organic inactivity it will greatly assist in the tonic treatment of the patient, while in the treatment of organic disease, it will afford great relief.

Phthisis is greatly benefited in its first stage by judicious application—the administration is certainly indicated on general principles.

To obtain the tonic and alterative effects of electro-static treatment, the wave current administered with the long spinal electrode and judicious use of long and friction sparks, are indicated.

*The local uses* as indicated by the physiological actions are many and most valuable.

*It relieves hyperemia and congestion.* The wave current, sparks and brush discharge are all instrumental in relieving such conditions.

No treatment can compare with these means in the treatment of sprains and bruises. If there has been no tearing of ligaments nor rupture of muscular fibers a very few treatments promptly cure, where months are required by other plans of treatment. Neglected and chronic cases, even when synovitis is present, yield to patient treatment by use of the wave current and sparks.

Many cases of acute and chronic rheumatism yield promptly, but others, in which a strong constitutional taint is present, resist for a longer time.

In all forms of neuritis the effects are as of a specific. If treated at the very outset, it is surprising how soon, what would otherwise be followed by months of suffering, is relieved, as is the case with sciatica and brachial neuritis. Do not follow the old rule and not apply electricty during the first week, but employ electro-static treatment at once, because it relieves hyperemia and congestion. The writer has now the record of ten cases each of less than two weeks' standing, cured within one week, and having treated upward of fifty cases, has yet to see one that could not be cured in a time relative to the chronicity. Malignant growths, tumors and necrosis are conditions that preclude the possibility of a cure.

Lumbago and myalgia are conditions usually promptly relieved and always curable by the wave current and sparks.

To acute congestions of the spinal cord or meninges, from any cause, prompt application of the wave current (never the constant) should be made because it diminishes congestion. We have seen this beautifully exemplified in anterior poliomyelitis in children.

The earlier the application is made, the more certain restoration will be complete. Wherever local congestion exists the wave current can be administered fearlessly and with hope if some specific process is not at work.

*It sedates pain and nervous irritability.* The pains of neuritis and neuralgia disappear like magic, in most cases, beneath the metal electrode when the wave current is employed. Gastralgia is relieved by placing a large electrode over the epigastrium. Angina pectoris is relieved by lowering of arterial tension, due to the constitutional effect and local sedation. For the treatment of migraine there is no surer permanent relief if the course of treatment is not too short. The pains of locomotor ataxia are far more amenable to electro-static treatment than to any agent but morphine, and if treatment is faithfully followed the periods between attacks lengthen and in most cases they cease altogether.

*Insomnia,* if not of too long standing, is, as a rule, promptly relieved by evening administrations or during a course of treatment by the wave current or general electrization.

*It overcomes muscular spasm or contractions.* An acute muscular contraction (cramp) yields promptly to a brisk application of sparks. Convulsive tic, if not of long standing, will yield promptly to the wave current and sparks. *The occupation neuroses,* writer's cramp, etc., if treated early, are promptly cured, but long-standing cases are apt to be stubborn. In all cases of occupation neuroses, treat the neurasthenia, which is sure to be present. In cases of spasmodic dysmenorrhea, spasmodic stricture of the urethra, or neck of the bladder, rectum or vagina employ the wave current, using a metal electrode as a speculum, sound or catheter in the absence of special ones. Applications of the wave current to the extremity of *producing fatigue* are only indicated when a spastic contraction exists, as in unyielding cases of torticollis; even then it is as a rule not efficacious.

*The rubefacient effects of the brush discharge* have a more profound effect over congestion than a sinapism. The antiseptic effects of the brush discharge suggest its application to skin affections. The writer has met with some success from its employment in eczema, but probably not owing to its antiseptic properties.

The advanced electro-therapeutics stands upon a base so broad and a foundation so stable, with scientific facts to establish and physiological results to verify, that aside from the certain deductions of surgery, it is the most positive force in materialistic medicine. This being a fact, three things are demanded: its general recognition, its place in the medical course of every medical college in the country, and its prescription by every physician. Suffering humanity deserves the best that the profession can give. The man who is not judiciously progressive in medicine stands in the way of his patient's best interest. To acquire the knowledge and technique necessary to make successful applications of electricity is not so difficult as is supposed, but if to be done to obtain satisfactory results requires some training. The uses of the constant current require close attention to measure of the quantity of the current adapted to the case and skill in manipulation, and the uses of static electricity require tact in managing the patient, knowledge of the parts and care of the machine, operative technique, choice of the form of application for a given condition, duration of treatment, dosage necessary to obtain results, and careful regulation of treatments as to frequency, observing the principle of bridging.

If those not well-informed will investigate, and those better informed than the writer will set the subject right before the profession, it will be of profit to humanity.

*Atlanta, Ga.*

